# Identifying Rare Variant Associations in Admixed Populations

**DOI:** 10.1038/s41598-019-41845-3

**Published:** 2019-04-01

**Authors:** Huaizhen Qin, Jinying Zhao, Xiaofeng Zhu

**Affiliations:** 1 Department of Epidemiology, College of Public Health and Health Professions and College of Medicine, University of Florida, Gainesville, FL 32611 USA; 2 Department of Global Biostatistics and Data Science, Tulane University School of Public Health and Tropical Medicine, 1440 Canal Street, New Orleans, LA 70112 USA; 3 Department of Population and Quantitative Health Sciences, Case Western Reserve University School of Medicine, 10900 Euclid Avenue, Cleveland, Ohio, 44106 USA

## Abstract

An admixed population and its ancestral populations bear different burdens of a complex disease. The ancestral populations may have different haplotypes of deleterious alleles and thus ancestry-gene interaction can influence disease risk in the admixed population. Among admixed individuals, deleterious haplotypes and their ancestries are dependent and can provide non-redundant association information. Herein we propose a local ancestry boosted sum test (LABST) for identifying chromosomal blocks that harbor rare variants but have no ancestry switches. For such a stable ancestral block, our LABST exploits ancestry-gene interaction and the number of rare alleles therein. Under the null of no genetic association, the test statistic asymptotically follows a chi-square distribution with one degree of freedom (1-df). Our LABST properly controlled type I error rates under extensive simulations, suggesting that the asymptotic approximation was accurate for the null distribution of the test statistic. In terms of power for identifying rare variant associations, our LABST uniformly outperformed several famed methods under four important modes of disease genetics over a large range of relative risks. In conclusion, exploiting ancestry-gene interaction can boost statistical power for rare variant association mapping in admixed populations.

## Introduction

Admixture is an omnipresent evolutionary force in complex disease genetics of recently admixed populations. Admixture mapping locates genomic segments that harbor causal alleles with distinct ancestral frequencies through admixture linkage disequilibrium (ALD). It has been successfully applied to locate genetic variants for a range of diseases and traits, e.g., hypertension^1,2^, type 2 diabetes^3,4^, obesity^5,6^ and Alzheimer’s dementia^7^. Systematic reviews of admixture mapping approaches can be found in the literature^8–10^. Genome-wide association studies (GWASs) have proven successful in identifying individual common genetic variants associated with common diseases and traits^11^. In the era of GWASs, admixture mapping has become a useful compliment for identifying common variant associations^12^. Several hybrid methods for combining the genotype and allele ancestry at a single-nucleotide polymorphism (SNP) have been developed^13–15^. These methods may claim variants which are in ALD with the true causal variants but not associated with the phenotype in any ancestral population. ALD may extend for substantial distances^16–18^. Qin and Zhu^12^ proposed a two-stage fine mapping method to first identify candidate local genomic segments and then identify individual variants responsible for the admixture mapping evidence.

The common variants identified in GWASs merely explain a small proportion of the heritability^19^, leading to many explanations of the ‘missing’ heritability^20–23^. A potential source of the missing heritability is the contribution of rare variants^24,25^. Evidenced by deep sequencing studies^26–28^, rare variants may have stronger effects on complex diseases than do common variants. Multiple methods have been developed for identifying rare variant associations. Collapsing methods, e.g., the CAST^29^ and the CMC^30^, utilize the number of rare alleles in a gene for each individual to enrich association information. The SDWSS^31^ scales SNPs in a test set by their minor allele frequencies in unaffected individuals. It utilizes a Wilcoxon type statistic to aggregate information and assesses the significance by permutation. The VT method^32^ utilizes the maximum of the test statistics over all allele-frequency thresholds. All these methods implicitly assume that all effects have an identical direction. To combine the effects of opposite directions, the data-adaptive sum test^33^ incorporates the signs of the observed effects into the CAST, whereas the C-alpha method^34^ and the SKAT^35^ test for genetic variance component. In particular, two methods have been proposed to combine the effects of different sizes and opposite directions. The ORWSS^36^ scales SNP wise numbers of minor alleles by the logarithms of amended odds ratios in the 2 × 2 tables of disease status by allele states. The EREC method^37^ scales SNP wise numbers of minor alleles by the estimated regression coefficients. When families are available, incorporating linkage evidence in rare variants analysis has also been developed^38–41^.

However, these existing methods may be underpowered for identifying rare variant associations in admixed populations, because they do not explicitly exploit the association information conveyed by local ancestries, particularly, ancestry-gene interaction. An admixed population and its ancestral populations often bear different burdens of complex diseases, partially due to the ancestral discrepancies in causal alleles, allele frequencies, and effects. Within a chromosomal block harboring causal alleles, an affected admixed individual may have an increased probability of inheriting alleles from the ancestry population of higher disease prevalence^12,42^. Common variants with different ancestral frequencies are correlated to their ancestries^43,44^ which provide non-redundant association information^12–15^. We hypothesize that this argument holds for rare variants. Single rare variant association testing has unacceptably limited power since only a small portion of study individuals carry the rare allele.

In this report, we will illustrate the utility of explicitly exploiting local ancestries and genotypes together for rare variant associations. For simplicity, we aim to identify stable ancestral blocks harboring rare variants. For each person, all the SNPs within such a block share an identical ancestry. We propose a heuristic local ancestry boosted sum test (LABST). In a stable ancestral block, our test statistic combines the sum of SNP wise numbers of rare mutations and the ancestry-gene interaction. We mathematically prove that the LABST statistic asymptotically follows a chi-square distribution with 1-df if the test block is not associated with the disease. In extensive simulations, our LABST appropriately controlled type I error rates at preset nominal levels, indicating the ideal accuracy of the asymptotic approximation. Under various multiple rare variant disease modes with a large range of relative risks, our LABST were uniformly more powerful than the benchmark CAST as well as the sophisticated SDWSS and ORWSS. The LABST is a heuristic method designed for unrelated cases and controls. It can be extended to incorporate informative weights, to accommodate covariates and to allow for multiple groups of rare variants.

## Methods

In an admixed population of two ancestral populations, let a test chromosomal block contain $L$ rare variants, i.e., the minor allele frequencies (MAFs) < 2%^45^. For *n* unrelated individuals from the admixed population, let *y*_*i*_ be disease status of individual *i* (*y*_*i*_ = 1, if individual *i* is affected; =0, if unaffected), *G*_1_ = {*i*: *y*_*i*_ = 1} and *G*_0_ = {*i*: *y*_*i*_ = 0} be the index sets of affected and unaffected individuals, respectively. Let *g*_*ij*_ denote the number of minor alleles carried by individual *i* at SNP *j*, ${s}_{i}=\sum _{j=1}^{L}\,{g}_{ij},$ and ***s*** = [*s*_1_, …, *s*_*n*_]. Let the test block be stable in terms of variant wise ancestries. In other words, we assume that each block wide haplotype of each individual is inherited entirely from one of the two ancestral populations without any ancestry crossover points. Under such an assumption, all the *m* SNPs within the block share identical ancestry. We define ***a*** = [*a*_1_, …, *a*_*n*_], where *a*_*i*_ denotes the number of ancestries on individual *i* inherited from the ancestral population of the higher disease prevalence (due to the larger risk haplotype frequency). Let *α* be the nominal significance level of a test for block-based associations.

### The proposed LABST

For each individual *i*, we define *u*_*i*_ = (1 + *a*_*i*_)*s*_*i*_ to combine ancestry-gene interaction *a*_*i*_*s*_*i*_ with *s*_*i*_. We define a Welch type *t* statistic

1 $${W}_{e}=\frac{{\bar{u}}_{1}-{\bar{u}}_{0}}{\sqrt{{\hat{\sigma }}_{1}^{2}/{n}_{1}+{\hat{\sigma }}_{0}^{2}/{n}_{0}}},$$

where ${\bar{u}}_{1}=\sum _{i\in {G}_{1}}\,{u}_{i}/{n}_{1}$, ${\hat{\sigma }}_{1}^{2}=\sum _{i\in {G}_{1}}\,{u}_{i}^{2}/{n}_{1}-{\bar{u}}_{1}^{2}$, and ${\bar{u}}_{0}^{2}$ and ${\hat{\sigma }}_{0}^{2}$ are likewise defined using the u-scores of *n*_0_ unaffected individuals. We use ${W}_{e}^{2}$ to measure the association between the test block and the disease status. Let *n*_1_/*n*_0_ converge to a finite positive constant *τ* when both *n*_0_ and *n*_1_ increase, e.g., *τ* = 1 if *n*_1_ = *n*_0_. If the test block is not associated with the disease status, then the statistic ${W}_{e}^{2}$ converges in distribution to $\xi  \sim {\chi }_{1}^{2}$, the chi-square distribution with 1-df (see Appendix A for a mathematical proof). Thus, we compute the *p*-value of ${W}_{e}^{2}$ as $P\mathop{=}\limits^{{\rm{def}}}{\rm{\Pr }}(\xi  > {W}_{e}^{2})$ and claim significance when *P* < *α*, the preset nominal significance level.

### Existing methods

Most existing rare variant association methods exploit genotypes without explicitly capitalizing on ancestry-gene interactions. In such methods, the genotypic score of individual *i* is defined as ${x}_{i}=\sum _{j=1}^{L}\,{w}_{j}{g}_{ij},$ where *w*_*j*_ is a SNP wise weight. The simplest weight is *w*_*j*_ ≡ 1 for all the *L* SNPs as in the CAST^29^. For this universal weight, *x*_*i*_ collapses to *s*_*i*_, and a benchmark 1-df statistic is constructed by replacing *u*_*i*_’s in our LABST with the *s*_*i*_’s.

The SDWSS^31^ weighs a SNP using its minor allele frequency in unaffected individuals among whole-sample individuals. At the *j*^th^ SNP, ${w}_{j}=1/\sqrt{n{q}_{j}(1-{q}_{j})},$ where *q*_*j*_ = (1 + *m*_*j*_)/(2 + 2*n*_0_), and *m*_*j*_ is the number of minor alleles at the SNP over the *n*_0_ unaffected individuals. Whole-sample individuals are ranked according to the *x*_*i*_ scores and a rank sum $x=\sum _{i\in {G}_{1}}\,{\rm{rank}}({x}_{i})$ is defined. Let ${x}_{1}^{\ast }$, …, $\,{x}_{k}^{\ast }$ be the rank sums based on *k*(=1,000) permutations of disease status, $\hat{\mu }$ and $\hat{\sigma }$, be their mean and standard deviation, respectively. The standardized score is defined as *z* = (*x*−$\hat{\mu }$)/$\hat{\sigma }$ and the *P* value of *z* is computed according to the standard normal distribution.

The weighting scheme in the SDWSS favors the disease-associated mutations with very low frequencies. As acknowledged by its authors, however, this scheme may reduce the power to detect the disease-associated mutations with higher frequencies. The SDWSS is based on the implicit assumption^32^ that $\mathrm{log}({{\rm{OR}}}_{j})\propto 1/\sqrt{{q}_{0j}(1-{q}_{0j})}$, where OR_*j*_ is the odds ratio in the 2 × 2 table of disease status by the allele at SNP *j*, and *q*_0*j*_ is the MAF in the controls. Thus, Feng *et al*.^36^ proposed the ORWSS to jointly analyze rare and common variants. This method keeps all the steps of the SDWSS except for the weighting scheme. In the ORWSS, a SNP is weighted by the logarithm of the amended odds ratio^46^ in the 2 × 2 table of allele by disease status. The amended odds ratio proves a useful remedy for handling potential empty cells in SNP-wise tables.

### Type I error rate inflation factor

Often, a conservative method tends more likely to miss true associations whereas a liberal method tends more likely to claim false positives. A valid powerful method should accurately control the type I error rate at each preset nominal level. Herein, we propose and use type I error rate inflation factor (TIERIF) to measure how accurately a method controls type I error rate. For a given nominal level *α*, we define the TIERIF of a method as *γ*_*α*_ = *τ*_*α*_/*α*, where *τ*_*α*_ is the probability that the method rejects the null hypothesis. If *γ*_*α*_ = 1, then the method is able to controls type I error rate at the given nominal level *α*. If *γ*_*α*_ is substantially smaller than 1, then the method is overly conservative. If *γ*_*α*_ is substantially larger than 1, then the method is overly liberal.

Usually, it is intractable to mathematically formulate the TIERIF of a sophisticated method. In addition, it is hard to tell what a TIERIF is unacceptably ‘small’ or ‘large’. Herein, we propose an empirical method to estimate this quantity and tell how small (large) is too small (large). Specifically, we define ${\hat{\gamma }}_{\alpha }={\hat{\tau }}_{\alpha }/\alpha$ as an estimator of *γ*_*α*_, where $\,{\hat{\tau }}_{\alpha }$ is the frequency that the method claims significance over *R* simulation replications generated under the null hypothesis of no association. As *R* increases, ${\hat{\gamma }}_{\alpha }$ converges in probability to *γ*_*α*_, and $\sqrt{R\alpha }({\hat{\gamma }}_{\alpha }-{\gamma }_{\alpha })/\sqrt{1-\alpha }$ converges in distribution to a standard normal variable (see Appendix B for a mathematical proof). Therefore, if the method properly controls type I error rate at *α*, then ${\hat{\gamma }}_{\alpha }$ concentrates with probability 95% between

2 $$L{B}_{\alpha }=1-1.96\sqrt{(1-\alpha )/(R\alpha )}$$

and

3 $$U{B}_{\alpha }=1+1.96\sqrt{(1-\alpha )/(R\alpha )}.$$

Under the null of no genetic association, the concentration interval [*LB*_*α*_, *UB*_*α*_] is the shortest among all the intervals [*LB*, *UB*] such that $\mathop{\mathrm{lim}}\limits_{R\to \infty }{{\rm{\Pr }}}_{0}(LB\le {\hat{\gamma }}_{\alpha }\le UB)=0.95$ (Appendix B). A method is called to be overly conservative if ${\hat{\gamma }}_{\alpha } < L{B}_{\alpha }$. Likewise, a method is called to be overly liberal if ${\hat{\gamma }}_{\alpha } > U{B}_{\alpha }$.

## Simulation Designs

For method comparisons, we simulated an admixture using the rare variants with the frequency-spectrums of two natural populations. In the simulated admixture, block-wide haplotypes were inherited from the two ancestry populations. Four disease genetic modes were considered, including the dominant, additive, recessive, and multiplicative modes. Under each disease genetic mode, the disease status of an admixed individual was determined by the penetrance conditioning on block-wide risk haplotypes other than individual risk alleles.

### Admixture

**Figure 1:**
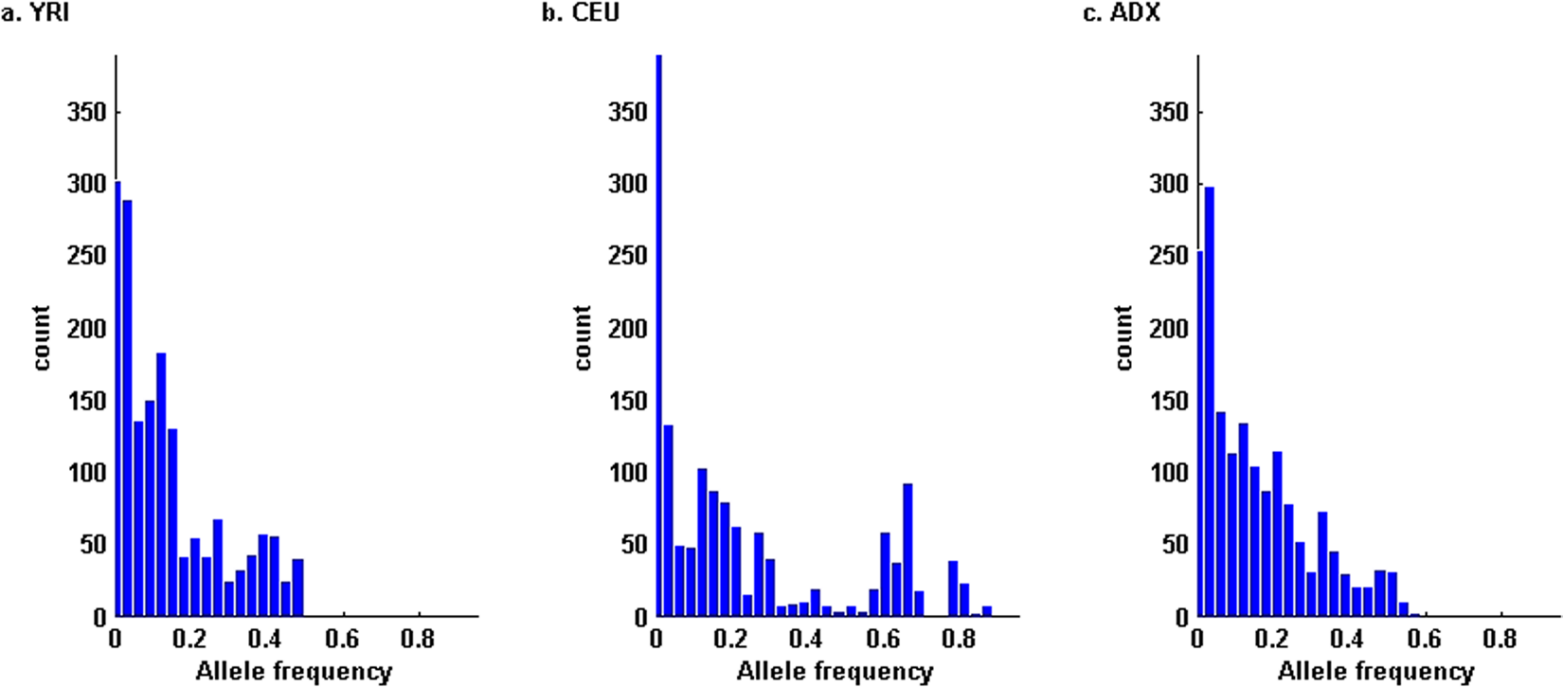
Population wise distributions of the reference alleles at 1,693 SNPs in ENr113.4q26. (**a**) In the YRI haplotype data, all the reference alleles are of frequencies *f*_*YRI*_ ≤ 0.5. (**b**) In the CEU haplotype data, the reference alleles at 1,373 SNPs are of frequencies *f*_*CEU*_ ≤ 0.5. (**c**) In the simulated admixture, the reference alleles at 295 SNPs are rare (0.001 < *f*_*ADX*_ < 0.02).

To simulate a two-way admixture, we downloaded the genotype data of region ENr113.4q26 from the ENCODE project Consortium^47^. Applying the Beagle software^48^, we separately inferred 180 CEU (Centre d’Etude du Polymorphisme Humain in Utah, USA) and 180 YRI (Yoruban in Ibadan, Nigeria) haplotypes over the ENr113.4q26 region. Details on the haplotype deconvolution have been described previously^36^. Across the 360 inferred haplotypes, we observed 1,693 SNPs. At each of the region-wide SNPs, we chose the minor allele in the YRI haplotype data (*f*_*YRI*_ ≤ 0.5) as the reference allele (Fig. 1a). In the CEU haplotype data, the reference alleles at 1,373 SNPs are of frequencies *f*_*CEU*_ ≤ 0.5, whereas at the other 320 SNPs, *f*_*CEU*_ > 0.5 (Fig. 1b). Based on our previous association study on African Americans^43^, we adopted *ω* = 0.8 vs. *ϖ* = 0.2 as YRI-CEU admixture weights. To ‘genotype’ one admixed individual in the ENr113.4q26 region, we randomly chose one and another haplotype from the YRI or CEU haplotypes with probabilities *ω* vs. *ϖ*. In this simulated admixture, the frequencies of reference alleles at the 1,693 SNPs (*f*_*ADX*_ = *ωf*_*YRI*_ + *ϖf*_*CEU*_) range from 0.0011 to 0.5722 (Fig. 1c), where 295 SNPs are of *f*_*ADX*_ < 0.02, satisfying the conventional criterion of rare variants^45^.

### Causal haplotypes and ancestries

From the 254 SNPs with *f*_*ADX*_ < 0.015, we randomly selected 23 SNPs as deleterious allele carriers (Table 1). The minor alleles at these 23 SNPs served as deleterious alleles. The deleterious alleles appear at 32 YRI and 4 CEU haplotypes, respectively. These 36 haplotypes served as risk haplotypes. The proportions of risk haplotypes in YRI and CEU haplotype data sets are ${p}_{YRI}=\frac{8}{45}\approx 0.1778$ and ${p}_{CEU}=\frac{1}{45}\approx 0.0222$, respectively. Thus, the proportion of risk haplotypes in the simulated admixture is

4 $${p}_{ADX}={p}_{YRI}\omega +{p}_{CEU}\varpi =\frac{8}{45}\times \frac{4}{5}+\frac{1}{45}\times \frac{1}{5}=\frac{11}{75}\approx \mathrm{0.1467.}$$

For each admixed individual, we let *H* be number of risk haplotypes and let *a* be the number of YRI haplotypes. Table 2 presents Pr(*H*, *a*), the joint probability mass of (*H*, *a*), where *q*_(⋅)_ = 1 − *p*_(⋅)_ for YRI, CEU and ADX, respectively. In this simulated admixture, the coefficient of correlation between *H* and *a* is

5 $$\rho =\frac{\sqrt{\varpi \omega }({p}_{YRI}-{p}_{CEU})}{\sqrt{(\omega {p}_{YRI}+\varpi {p}_{CEU})(\omega {q}_{YRI}+\varpi {q}_{CEU})}}=\frac{7}{12\sqrt{11}}\approx \mathrm{0.1759.}$$

**Table 1:** The distribution of the frequencies of deleterious alleles.

SNPs	Base pair positions	Reference alleles	Frequencies		
			*f*_*YRI*_	*f*_*CEU*_	*f*_*ADX*_
rs10020425	118710926	C	1/180	0	0.004444444
rs4834616	118781194	G	1/90	1/180	0.01
rs17866922	118799469	T	1/180	0	0.004444444
rs17866231	118811782	G	1/180	0	0.004444444
rs17869437	118846471	A	1/90	0	0.008888889
rs17866969	118860941	T	1/60	0	0.013333333
rs17866219	118861303	G	1/180	0	0.004444444
rs11931936	118880832	A	1/60	0	0.013333333
rs17869443	118891959	G	1/180	0	0.004444444
rs13138706	118901935	A	1/90	0	0.008888889
rs17865249	118942421	A	1/180	0	0.004444444
rs17867496	118943543	G	1/180	1/60	0.007777778
rs17862037	118948929	C	1/180	1/60	0.007777778
rs17868674	118950202	C	1/180	0	0.004444444
rs17875131	118953676	C	1/180	1/60	0.007777778
rs11562912	118958053	C	1/60	0	0.013333333
rs17867082	118986061	C	1/60	0	0.013333333
rs11945465	119041637	C	1/90	0	0.008888889
rs11929977	119118756	C	1/180	0	0.004444444
rs17867208	119122424	A	1/60	0	0.013333333
rs17869338	119143264	T	1/180	0	0.004444444
rs17866812	119152274	T	1/60	0	0.013333333
rs17867083	119185964	T	1/90	0	0.008888889

**Table 2:** Generic probability mass function of (***H***, ***a***) in the simulated admixture.

	*a* = 0	*a* = 1	*a* = 2	Pr(*H*)
*H* = 0	${q}_{CEU}^{2}{\varpi }^{2}$	2*q*_*CEU*_*q*_*YRI*_*ϖω*	${q}_{YRI}^{2}{\omega }^{2}$	${q}_{ADX}^{2}$
*H* = 1	2*p*_*CEU*_*q*_*CEU*_*ϖ*^2^	2 (*q*_*CEU*_*p*_*YRI*_ + *p*_*CEU*_*q*_*YRI*_)*ϖω*	2*p*_*YRI*_*q*_*YRI*_*ω*^2^	2*p*_ADX_*q*_ADX_
*H* = 2	${p}_{CEU}^{2}\,{\varpi }^{2}$	2*p*_*CEU*_*p*_*YRI*_*ϖω*	${p}_{YRI}^{2}{\omega }^{2}$	${p}_{ADX}^{2}$
Pr(*a*)	*ϖ* ^2^	2*ϖω*	*ω* ^2^	

### Modes of disease genetics

Let *y* be the disease status of an admixed individual (=1, if affected; =0, if unaffected). Let *f*_*H*_ = Pr(*y* = 1|*H*) be the penetrance for a given *H* value (=0, 1, or 2). Then the disease prevalence *κ* = Pr(*y* = 1) can be formulated as

6 $$\kappa =\sum _{H}\,{f}_{H}\,{\rm{\Pr }}(H).$$

Let *RR* = *f*_2_/*f*_0_ be relative risk. Then, ${f}_{1}={f}_{0}\cdot RR$ for dominant mode, ${f}_{1}=\frac{1}{2}{f}_{0}\cdot (1+RR)$ for additive mode, ${f}_{1}={f}_{0}\cdot \sqrt{RR}$ for multiplicative mode, and *f*_1_ = *f*_0_ for recessive mode. Under each mode, ${\rm{\Pr }}(y,a|H)={\rm{\Pr }}(y|H)$ ${\rm{\Pr }}(a|H)$, namely, the disease status is independent of local ancestry *a* given haplotype *H*. It follows that

7 $${\rm{\Pr }}(H,a|y)=\frac{{\rm{\Pr }}(H,a){\rm{\Pr }}(y|H)}{{\rm{\Pr }}(y)}$$

for an arbitrary (*H*, *a*, *y*). Setting *y* = 0 in Eq. (7) yielding the joint probability mass of (*H*, *a*) in unaffected subpopulation:

8 $$Pr(H,a|y=0)=\frac{Pr(H,a)(1-{f}_{H})}{1-\kappa }.$$

Setting *y* = 1 in Eq. (7) yielding the joint probability mass of (*H*, *a*) in affected subpopulation:

9 $${\rm{\Pr }}(H,a|y=1)=\frac{\Pr (H,a){f}_{H}}{\kappa }.$$

Eqs (8) and (9) and Table 2 are necessary and sufficient to mathematically formulate the Pearson coefficient of the correlation between *H* and *a* in the entire affected subpopulation ${\rho }_{1}\mathop{=}\limits^{{\rm{def}}}{\rm{corr}}(H,a|y=1)$ and that in the entire unaffected subpopulation $\,{\rho }_{0}\mathop{=}\limits^{{\rm{def}}}{\rm{corr}}(H,a|y=0)$.

### Simulation configurations

**Figure 2:**
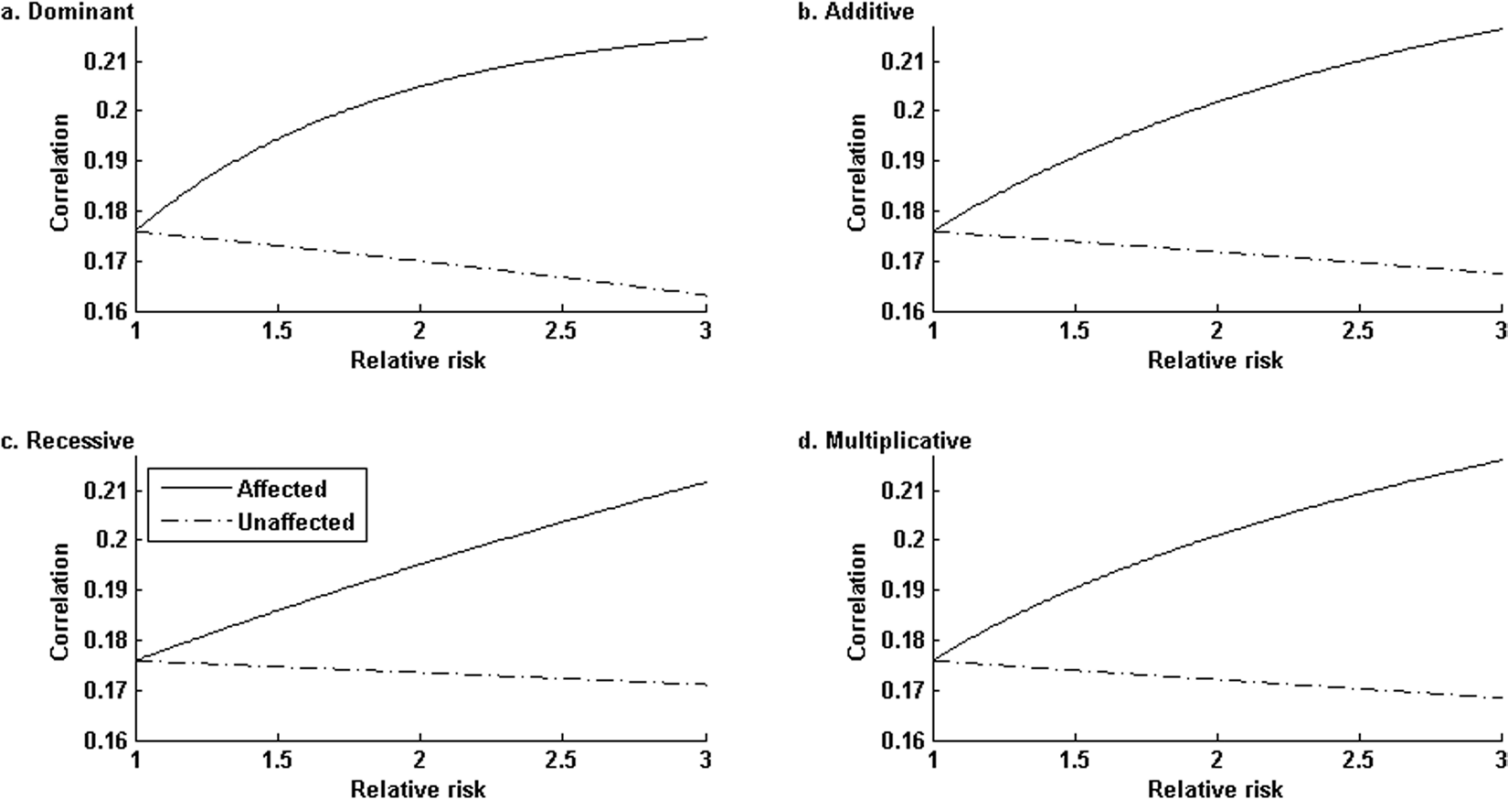
The trends of correlation between the numbers of causal haplotypes and ancestries under four modes of disease genetics. The correlation curves in each panel were generated by fixing *f*_0_ = 0.1 and varying (*f*_1_, *f*_2_) according to the underlying genetic modes. Generically, as relative risk increases from 1 to 3, the coefficient of correlation between *H* and *a* in affected group corr(*H*, *a*|*D* = 1) increases from 0.1759, whereas that in unaffected group decreases from 0.1759. The dominant mode shows the largest ratio of corr(*H*, *a*|*D* = 1) to corr(*H*, *a*|*D* = 0), followed in turn by the additive, multiplicative and recessive modes.

Using Table 3, we numerically computed *ρ*_1_ and *ρ*_0_ values for *f*_0_ = 0.1 and each *RR* under each of the four disease genetic modes (Fig. 2). Under all the four modes, *ρ*_1_ increases and *ρ*_0_ decreases from 0.1759 as *RR* increases from 1 (no genetic association) to 3. The dominant mode shows the largest ratio *ρ*_1_/*ρ*_0_, followed in turn by the additive mode, the multiplicative mode, and the recessive mode. Of note, *f*_0_ = *f*_1_ = *f*_2_ = *κ* under the null hypothesis of no genetic association. We acknowledge that prevalence (*κ*) varies for different diseases in an admixed population. For example, about 10% African Americans suffer from lifetime major depressive disorder^49^, whereas about 2.7% African American suffer from dementia^50^. In our simulations, we fixed *f*_0_ = 0.1 as a reference value to inspect the type I error rate and power patterns of different association methods with respect to different disease modes, relative risks, sample sizes, and nominal significance levels.

**Table 3:** Specific probabilities of (***H***, ***a***) used in the simulation*.

	*a* = 0	*a* = 1	*a* = 2	Pr(*H*)
*H* = 0	0.03824198	0.25726420	0.43267160	0.7281778
*H* = 1	0.00173827	**0.0614716**	**0.1871012**	0.2503111
*H* = 2	0.00000198	**0.0012642**	**0.0202272**	0.0215111
Pr(*a*)	0.04	0.32	0.64	

*Under this specific joint distribution of (*H*, *a*), the variance of (1 + *a*)*H* is Var[(1 + *a*)*H*] = 2.019955.

For each *RR* value under each mode, at each replication we simulated region wide genotypes and ancestries of *n*_1_ affected and *n*_0_ unaffected individuals from the admixed population. For each specific scenario, we adopted sample sizes *n*_1_ = *n*_0_ = 500 and then *n*_1_ = *n*_0_ = 2,000 to inspect the impacts of sample sizes on power levels and type I error rates of the methods under comparison. These sample sizes are realistic in that they reflect the scales of recent deep sequencing studies in African Americans. For example, 489 Alzheimer’s cases and 472 controls were sequenced a target sequencing study on Alzheimer’s disease^51^. The Jackson Heart Study^52^ has deeply sequenced more than 3400 African Americans.

To accurately evaluate the TIERIFs of the methods, we simulated 10^8^ replications of genotypes and ancestries by setting *RR* = 1 under each of the four disease modes. This number of replications is sufficient and necessary for evaluating type I error rates of gene-based tests at nominal genome-wide significance level (2.5 × 10^−6^). For power comparisons, we generated 20,000 replications for each given *RR* value (>1) under each of the four disease modes. This number of replications would be sufficient for reliably inspecting power patterns.

The ORWSS was designed to accommodate both rare and common variants. Thus, for this method, we used all the region wide variants to compute the weighted-sum of genotypic scores. The SDWSS have been observed to reduce statistical power when more neutral common variants are included into the test statistic. Intuitively, the other two methods will also reduce statistical power if common neutral variants are included. In our power comparisons, therefore, we used the conventional threshold 0.02 to choose rare variants^36^ to perform the other three methods. In the simulated admixture, the reference alleles at 295 SNPs proved rare (*f*_*ADX*_ < 0.02). Hence, we used the numbers of minor alleles at these 295 SNPs to compute the sum scores in the CAST and our LABST as well as the weighted-sum score in the SDWSS.

## Results

### Type I error rates

**Figure 3:**
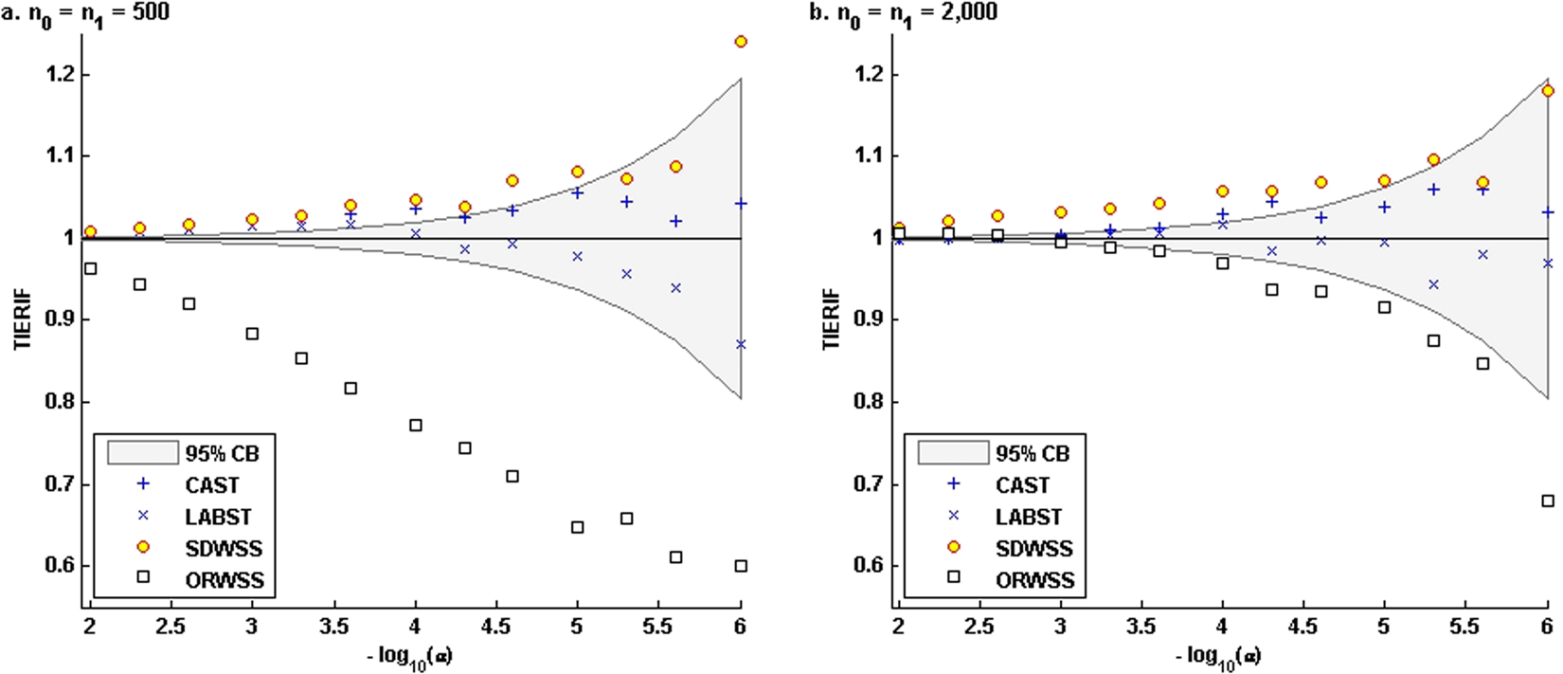
Empirical TIERIFs of the four methods based on different sample sizes and various nominal levels. In each panel, we generated 10^8^ replications of region wide genotypes and ancestries of the specified numbers of affected and unaffected admixed to evaluate the TIERIF of each method at each nominal level. In the ORWSS, we used all the 1,693 variants to compute the individual weighted-sums of genotypic scores. In the other methods, we used the 295 variants of *f*_*ADX*_ < 0.02 to compute the individual sums/weighted-sums of genotypic scores. The LABST and the CAST accurately controlled the type I error rates. The SDWSS appeared overly liberal. The ORWSS appeared over conservative, particularly for the smaller samples.

Figure 3 presents the TIERIFs of the four methods. For both sets of sample sizes, the CAST and our LABST well control type I error rates for various nominal levels across interval [10^−6^, 0.05]. They do not inflate or deflate type I error rates. Their TIERIF curves consistently concentrate around 1 and within the 95% concentration band (CB). The SDWSS appears overly liberal and the type I error rate inflation is quite robust to the increase in sample size. Its TIERIF curves clearly break the upper bound of the 95% CB for both the smaller and the larger sample size settings. These results would suggest that the SDWSS suffers a systematic bias in calibrating the tail probability of its test statistic. In contrast, the ORWSS appears overly conservative. Its TIERIF curves clearly break the lower bound of the 95% CB, especially for the smaller sample sizes. It becomes essentially less conservative for the larger sample sizes. These results would suggest that the ORWSS better calibrates the tail probability of its test statistic for larger sample sizes.

### Power comparisons

**Figure 4:**
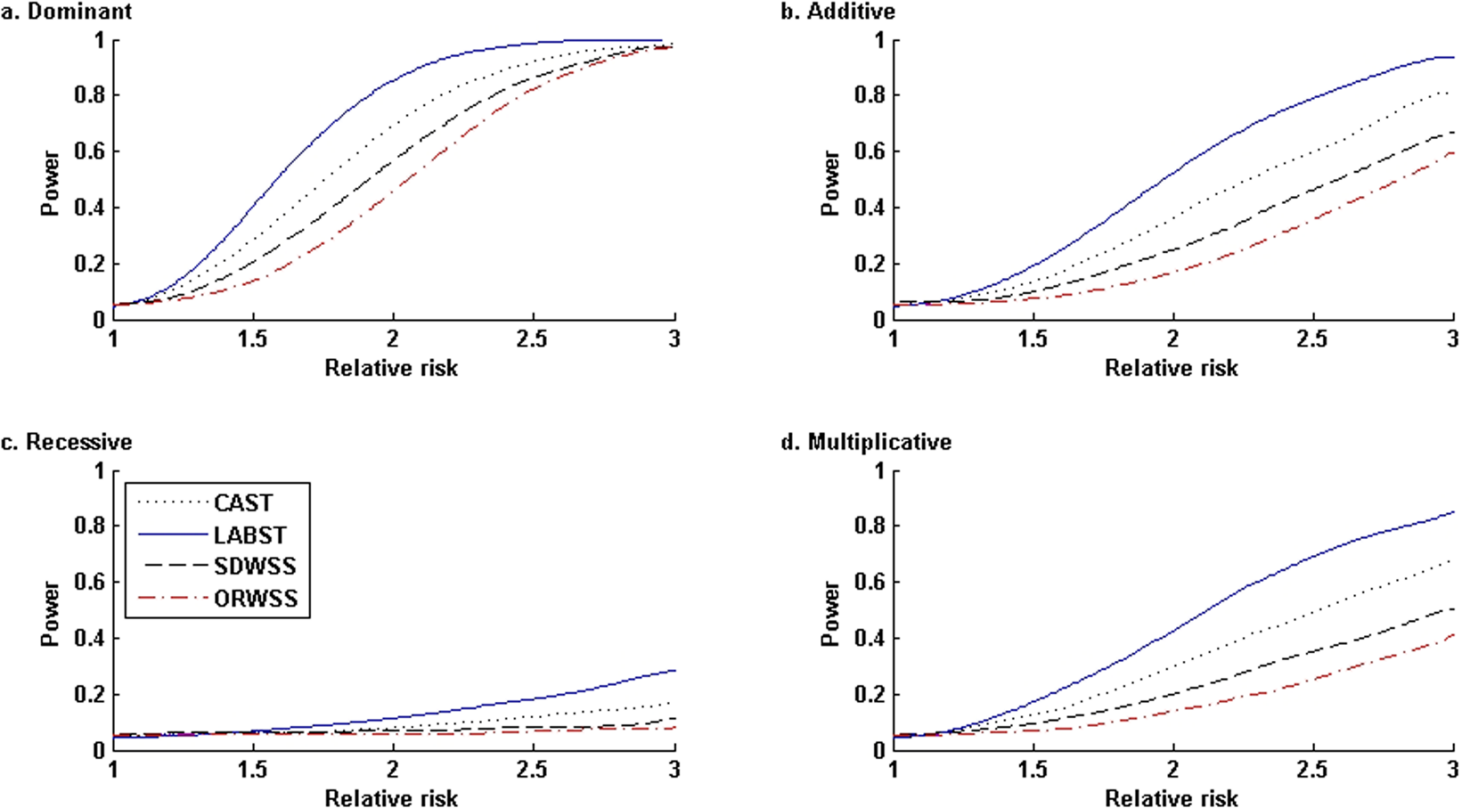
Power comparisons under four disease modes with various relative risks: *n*_0_ = *n*_1_ = 500, nominal level *α* = 0.05. In the simulated admixture, all the 23 risk allele frequencies are less than 0.015, and the cumulative risk haplotype frequency is 0.1467. Under each mode, at each relative risk, we evaluated the power of each method based on 20,000 simulated replications. In the ORWSS, we used all the 1,693 variants to compute the individual weighted-sums of genotypic scores. In all the other methods, we used the 295 variants of *f*_*ADX*_ < 0.02 to compute the individual sums/weighted-sums of genotypic scores.

Figure 4 presents the power comparisons under four disease genetic modes with sample sizes *n*_0_ = *n*_1_ = 500 and nominal level *α* = 0.05. Overall, each method performs the best at the dominant mode, followed in turn by the additive mode, multiplicative mode, and lastly, recessive mode. Under each disease genetic mode, our LABST performs the best for all relative risks, followed in turn by the CAST, the SDWSS, and the ORWSS. Exploiting an identical set of rare variants, the CAST uniformly outperforms the SDWSS. Since the SDWSS is robustly liberal, its power inferiority would be caused by the transformation of the weighted-sums of genotypic scores to ranks, which would lose information. For the moderate sample sizes, the ORWSS appears unacceptably conservative and lacks ability to effectively separate the true causal variants from the other variants.

**Figure 5:**
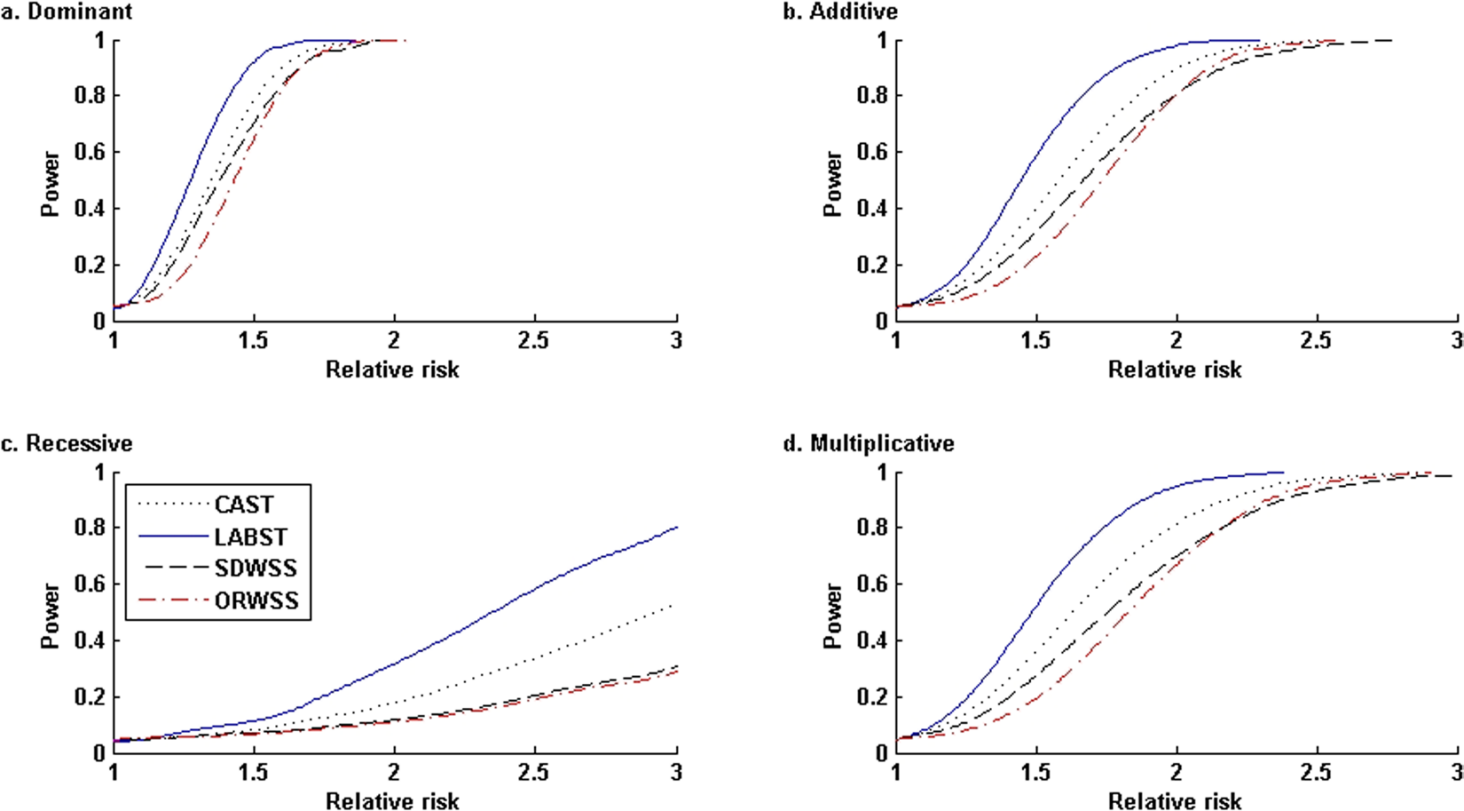
Power comparisons under four disease modes with various relative risks: *n*_0_ = *n*_1_ = 2,000, nominal level *α* = 0.05. In the simulated admixture, all the 23 risk allele frequencies are less than 0.015, and the cumulative risk haplotype frequency is 0.1467. Under each mode, at each relative risk, we evaluated the power of each method based on 20,000 simulated replications. In the ORWSS, we used all the 1,693 variants to compute the individual weighted-sums of genotypic scores. In all the other methods, we used the 295 variants of *f*_*ADX*_ < 0.02 to compute the individual sums/weighted-sums of genotypic scores.

Figure 5 presents the power comparisons when increasing sample size to *n*_0_ = *n*_1_ = 2,000 but keeping all the other parameters used in Fig. 4. All the methods show increased power across all the different disease genetic modes. The LABST keeps its uniform preference, followed by the CAST, which outperforms the SDWSS and the ORWSS. However, the ORWSS now outperforms the SDWSS for a wide range of relative risks under the dominant, additive, and multiplicative modes. Under the recessive mode, the SDWSS slightly outperforms the ORWSS for all the relative risks. When increasing the sample size, the ORWSS becomes much less conservative and better scales the causal SNPs especially when RR becomes relatively large.

**Figure 6:**
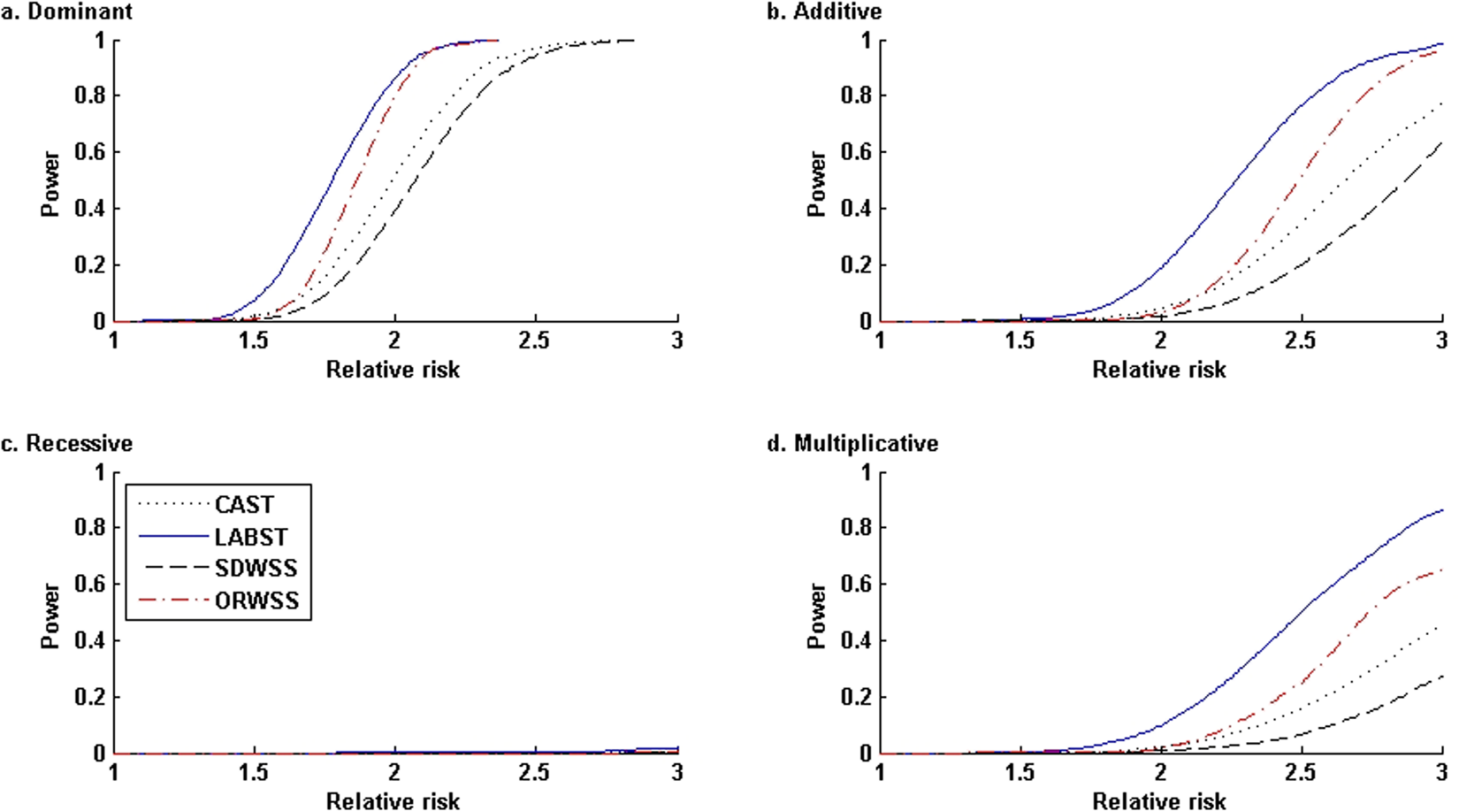
Power comparisons under four disease modes with various relative risks: *n*_0_ = *n*_1_ = 2,000, nominal level *α* = 10^−6^. In the simulated admixture, all the 23 risk allele frequencies are less than 0.015, and the cumulative risk haplotype frequency is 0.1467. Under each mode, at each relative risk, we evaluated the power of each method based on 20,000 simulated replications. In the ORWSS, we used all the 1,693 variants to compute the individual weighted-sums of genotypic scores. In all the other methods, we used the 295 variants of *f*_*ADX*_ < 0.02 to compute the individual sums/weighted-sums of genotypic scores.

Figure 6 presents the power comparisons when reducing the nominal level to *α* = 10^−6^ but keeping all the other parameters in Fig. 5. At this significance level, the LABST still outperforms the other methods across all the scenarios. Under the recessive mode, all methods have very low or no power with respect to various relative risks. Under the other three modes, the CAST is more powerful than the SDWSS but becomes less powerful than the ORWSS, whereas the ORWSS become the second best among our compared methods for a wide range of relative risks.

## Discussion

The primary objective of this report is to illustrate the utility of leveraging local ancestry for rare variant association analysis. We present the LABST to combine local ancestry of a test block with the sum of genotypic scores of block-wide rare variants. Under the null of no genetic association, we mathematically prove that the LABST statistic asymptotically follows the chi-square distribution of one degree of freedom. This explicit asymptotic null distribution enables us analytically compute the significance of each ancestry block. Under our extensive simulations, the LABST properly controls type I error rates at various preset nominal levels. These results indicate that the null distribution of the LABST statistic can be accurately approximated by the 1-df chi-square distribution. Based on our results, the permutation-based evaluations of significance in the SDWSS and ORWSS are not accurate enough for genome-wide scans for samples from admixed populations. The SDWSS tends to inflate type I error rates and the inflation appears robust to the changes of sample sizes. In other words, the SDWSS would suffer a systematic bias in calibrating the tail probability of its test statistic. The ORWSS appears severely conservative when the numbers of affected and unaffected individuals are moderate. Its conservativeness becomes less significant when the sample sizes are essentially increased. The conservativeness of the ORWSS would stem from the unideal stability and effectiveness of its weighting scheme.

In our simulations for the power comparisons, we hypothesize that certain haplotypes of some rare alleles are direct causal factors. This assumption allows for diverse SNP wise MAFs but does not necessarily mean that alleles with smaller MAFs have larger effect sizes. Under four disease genetic modes, the LABST are uniformly more powerful than the CAST, SDWSS, and ORWSS across all relative risks, sample sizes, and nominal levels investigated. Its power gain stems from explicit incorporation of the interaction between a gene and the local ancestry. The CAST is more powerful than the SDWSS uniformly across all our simulations, even though the SDWSS is robustly liberal. The SDWSS loses portion of information when transforming the original weighted-sums of numerical genotypic scores to Wilcoxon rank-sum statistic. As pointed out by Wilcoxon himself, ranks are not sufficient statistics^53^ and hence rank-sum test would not be the most powerful test. The superiority of the ORWSS to the CAST and the SDWSS varied across different disease genetic modes, relative risks, sample sizes, and nominal significance levels. Based on our results, the ORWSS would have limited utility for studies of small to moderate samples, whereas it would be useful for studies with large samples from a homogeneous population. Based on our results, a liberal method is not necessarily more powerful uniformly than a conservative method. The preference of a method depends on how effectively it can aggregate the association information of rare variants. Although derived from simulations on a particular region, all the conclusions are generalizable for an arbitrary admixed population with different ancestry-haplotype correlations between cases and controls. Such differences often stem from the different ancestral frequencies of the risk haplotypes, disease modes, and/or relative risks.

Our LABST can be extended to Hotelling’s two-sample *T*-squared test^54^ to jointly analyze multiple groups of variants when desired. Following the LABST, we can define *u*_*ij*_ as the integrative score of individual *i* in group *j*. For *d* groups, we write ***u***_*i*_ = (*u*_*i*1_, …, *u*_*id*_)′ as the *d* × 1 vector of integrative scores, ${\bar{{\boldsymbol{u}}}}_{0}=\sum _{i\in {G}_{0}}\,{{\boldsymbol{u}}}_{i}/{n}_{0}$, ${\bar{{\boldsymbol{u}}}}_{1}=\sum _{i\in {G}_{1}}\,{{\boldsymbol{u}}}_{i}/{n}_{1}$, and ***V*** = (*n*−2)^−1^ $[\sum _{i\in {G}_{1}}\,({{\boldsymbol{u}}}_{i}-{\bar{{\boldsymbol{u}}}}_{1})({{\boldsymbol{u}}}_{i}-{\bar{{\boldsymbol{u}}}}_{1})^{\prime} +\sum _{i\in {G}_{0}}\,({{\boldsymbol{u}}}_{i}-{\bar{{\boldsymbol{u}}}}_{0})({{\boldsymbol{u}}}_{i}-{\bar{{\boldsymbol{u}}}}_{0})^{\prime} ].$ We define Hotelling’s statistic as ${T}^{2}=({n}_{0}{n}_{1}/n)({\bar{{\boldsymbol{u}}}}_{1}-{\bar{{\boldsymbol{u}}}}_{0}){{\boldsymbol{V}}}^{-1}({\bar{{\boldsymbol{u}}}}_{1}-{\bar{{\boldsymbol{u}}}}_{0})$. The covariance matrix ***V*** converges in probability to a positively definite matrix as long as the integrative scores are not in co-linearity. The statistic *T* ^2^ converges in distribution to the chi-square distribution with *d* degrees of freedom if group set is not associated with the disease. In addition, informative group wise weights, if available, can be readily incorporated into the Hotelling’s *T* ^2^ test.

In this investigation, individual local ancestries were assumed to be known. In practice, local ancestries can be inferred from available genomic data. Several software packages, such as SABER^18^, HAPAA^55^, HAPMIX^56^, MULTIMIX^57^, CSVs^58^, and ELAI^59^, have been established for inferring local ancestries. These packages utilize available marker-wise genotypes of a target individual and the haplotypes/genotypes from certain ancestral panels. When dense SNPs are genotyped across the genome, the local ancestries can be highly accurately inferred. For each admixed individual, our LABST assumes that within a short block there is no ancestry crossover. This assumption is reasonable for haplotype blocks in ancestral populations^60,61^. Such haplotype blocks are of little evidence for historical recombination and much shorter than ALD regions. Gene based rare variant associations often fall in such blocks. In practice, it would be important to accommodate covariates (e.g., population structure variables, environmental factors). Let ***z***_*i*_ = [1, *z*_*i*1_, …, *z*_*ic*_]′ contain the covariates of individual *i* and let ***Z*** = [***z***_1_, …, ***z***_*n*_]′ be the whole sample covariates matrix. To adjust for the covariates, let ${e}_{i}={u}_{i}-{z}_{i}{\rm{^{\prime} }}{(Z{\rm{^{\prime} }}Z)}^{-1}Z{\rm{^{\prime} }}u$ for each individual *i*, where ***u*** = [*u*_1_, …, *u*_*n*_]′. It is clear that the vector of residuals ***e*** = [*e*_1_, …, *e*_*n*_]′ is orthogonal to ***Z***, that is, ***e***′***Z*** = 0. Replacing *u*_*i*_’s in the statistic *W*_*e*_ (Eq. 1) with *e*_*i*_’s is one way to adjust for covariates.

Like many existing methods, our LABST assumes that individuals are randomly recruited from a target admixed population. It will be instructive to develop particular integrative methods for other sampling schemes that enrich rare variants. For example, the individuals with extreme values of a quantitative trait are often recruited for sequencing studies. Under such a trait-oriented sampling scheme, the LABST is valid but its power would be improved by combining local ancestry with a direct quantitative association analysis that incorporates the sampling scheme. In addition, individuals can be selected according to a secondary sampling trait, which is conveniently and economically measured. Only for the recruited individuals, the values of the primary study trait are measured. For such a sampling scheme, we will develop novel effective methods to combine block wise ancestries and genotypes with multiple phenotypes for identifying pleiotropic genes. Currently, the LABST only works for a recent (several-generations) admixture of two ancestral populations with different genetic architectures, i.e., distinct causal allele frequencies and/or effects. One typical example is the current African American population, which suffers from disproportionately heavier burdens of multiple diseases^1–7^ than European Americans. The LABST can be extended to allow for multi-way admixtures such as Hispanic and Latino Americans. For example, it can be extended to a (*d* + 1)-way admixture by using Hotelling’s two-sample *T*-squared test with *d* degrees of freedom, which is similar to the above extension to combine multiple groups of variants.

## Supplementary information

Appendix
