# Supplementary material for: Identifying Rare Variant Associations in Admixed Populations
Source: Sci Rep. 2019 Apr 1;9:5458. doi: 10.1038/s41598-019-41845-3 (PMC6443736; doi:10.1038/s41598-019-41845-3)
Supplement: Supplementary file 1 — Appendix [file 41598_2019_41845_MOESM1_ESM.docx]

**Identifying Rare Variant Associations in Admixed Populations**

**Huaizhen Qin^1,2^, Jinying Zhao^1^, and Xiaofeng Zhu^3,*^**

^1^*Department of Epidemiology, College of Public Health and Health Professions and College of Medicine, University of Florida, Gainesville, FL 32611, USA*

^2^*Department of Global Biostatistics and Data Science, Tulane University School of Public Health and Tropical Medicine, 1440 Canal Street, New Orleans, LA 70112, USA*

^3^*Department of Population and Quantitative Health Sciences, Case Western Reserve University School of Medicine, 10900 Euclid Avenue, Cleveland, Ohio 44106, USA*

**Running Title: Rare variant analysis in admixed populations**

*Corresponding author:

Xiaofeng Zhu, Ph.D., Professor

Department of Population and Quantitative Health Sciences

Case Western Reserve University School of Medicine

10900 Euclid Avenue, Cleveland, Ohio 44106, USA

Phone: (216) 368-0201

E-mail: xxz10@case.edu

**Appendix A**

Under the null of no genetic association, $u_{i}$ is independent and identically distributed. Let $\mu$ and $\sigma^{2}$ be, respectively, the mathematical expectation and variance of$u_{i}$. Then equation ($1$) can be rewritten as

$$W_{e}=w_{1}\times\sqrt{n_{1}}\left( \bar{u}_{1}-\mu\right)+w_{0}\times\sqrt{n_{0}}\left( \bar{u}_{0}-\mu\right), (A1)$$

where $w_{1}=1/\sqrt{\hat{\sigma}_{1}^{2}+(\hat{\sigma}_{0}^{2}{n_{1}}/{n_{0}})}$ and $w_{0}=-1/\sqrt{\hat{\sigma}_{1}^{2}\left( {n_{0}}/{n_{1}} \right)+\hat{\sigma}_{0}^{2}}$. When both$n_{0}$ and $n_{1}$ increase, by Lindeberg-Lévy’s central limit theorem (Serfling^[1](#_ENREF_1)^, p.28),

$$\left[ \begin{matrix} \sqrt{n_{1}}\left( \bar{u}_{1}-\mu\right) \\ \sqrt{n_{0}}\left( \bar{u}_{0}-\mu\right) \end{matrix} \right]\underset{\to}{d.}\left[ \begin{matrix} \zeta_{1} \\ \zeta_{0} \end{matrix} \right]\sim\mathcal{N}_{2}\left( \left[ \begin{aligned} 0 \\ 0 \end{aligned} \right], \sigma^{2}\left[ \begin{matrix} 1 & 0 \\ 0 & 1 \end{matrix} \right] \right), (A2)$$

where “$\underset{\to}{d.}$” reads as “converges in distribution to”, and $\mathcal{N}_{2}\left( \cdot,\cdot\right)$ stands for bivariate normal distribution, and the independence (0 correlation) between $\zeta_{1}$ and $\zeta_{0}$ stems from the independence between cases and controls. By the strong law of large numbers (Serfling^[1](#_ENREF_1)^, p.69), both$\hat{\sigma}_{1}^{2}$ and $\hat{\sigma}_{0}^{2}$ converge to $\sigma^{2}$ almost surely. It follows that $w_{1}\to\omega_{1}=1/\sqrt{\left( 1+\tau\right)\sigma^{2}}$ and $w_{0}\to\omega_{0}=-/\sqrt{\left( 1+1/\tau\right)\sigma^{2}}$ almost surely, given that $n_{1}/n_{0}\to\tau$ when both$n_{0}\to\infty$ and $n_{1}\to\infty$. By Slutsky's theorem (Serfling^[1](#_ENREF_1)^, p.19), $W_{e}\underset{\to}{d.}\zeta=\omega_{1}\zeta_{1}+\omega_{0}\zeta_{0}$. Note that$\omega_{1}^{2}+\omega_{0}^{2}=1/\sigma^{2}$ and hence$\zeta\sim\mathcal{N}(0,1)$. Therefore, $W_{e}^{2}\underset{\to}{d.}\chi_{1}^{2}$ under the null.

**Appendix B**

For a given nominal level$\alpha$, let $\tau_{\alpha}$ be the probability that a method rejects the null of no association. For each simulation replication $i$, define where $B_{i}=1$if the method claims significance, and = 0 otherwise. Then $B_{i}\mathfrak{\sim B}\left( 1,\tau_{\alpha} \right)$, the Bernoulli distribution with success probability $\tau_{\alpha}$, and $\hat{\tau}_{\alpha}=\sum_{i=1}^{R} B_{i}/R$ is an unbiased estimator of $\tau_{\alpha}$ based on $R$ independent replications. By Lindeberg-Lévy’s central limit theorem (Serfling^[1](#_ENREF_1)^, p.28), $\sqrt{R}\left( \hat{\tau}_{\alpha}-\tau_{\alpha} \right)\underset{\to}{d.}\mathcal{N}\left( 0,1 \right)$ as $R\to\infty$. By Slutsky's theorem (Serfling[^1^](#_ENREF_1), p.19),

$$\frac{\sqrt{R\alpha}\left( \hat{\gamma}_{\alpha}-\gamma_{\alpha} \right)}{\sqrt{1-\alpha}}\underset{\to}{d.}\mathcal{N}\left( 0,1 \right). (B1)$$

Of note, $\gamma_{\alpha}=1$ if the method properly controls type I error rate at$\alpha$, namely, $\tau_{\alpha}=\alpha$. It follows that

$$z≝\frac{\sqrt{R\alpha}\left( \hat{\gamma}_{\alpha}-1 \right)}{\sqrt{1-\alpha}}\underset{\to}{d.}\mathcal{N}\left( 0,1 \right) (B2)$$

By ($B2$), when $R$ is large, $z$ can be taken as a sample (with size 1) from the standard normal distribution. Thus, the shortest 95% CI of $z$ for 0 is $[-1.96, 1.96]$ [^2^](#_ENREF_2)^,^[^3^](#_ENREF_3). It follows that $[{LB}_{\alpha}, {UB}_{\alpha}]$ is the shortest asymptotic CI of $\hat{\gamma}_{\alpha}$ for 1 under the null of no genetic association.

1 Serfling, R. J. *Approximation theorems of mathematical statistics*. Vol. 162 (John Wiley & Sons, 2009).

2 Boubakar, T., Lassina, D., Belco, T. & Abdou, F. The Shortest Confidence Interval for the Mean of a Normal Distribution. *International Journal of Statistics and Probability* **7**, 33 (2018).

3 Guenther, W. C. Shortest confidence intervals. *The American Statistician* **23**, 22-25 (1969).
